# Intimate partner psychological violence and children’s sleep difficulties up to 5 years of age: an ELFE birth cohort

**DOI:** 10.1093/eurpub/ckaf037

**Published:** 2025-04-04

**Authors:** Marion Bailhache, Sabine Plancoulaine, Fabienne El-Khoury, Olivier Leproux, Eloi Chazelas, Ramchandar Gomajee, Judith Van Der Waerden, Marie Aline Charles, Maria Melchior

**Affiliations:** Pole de pediatrie, Place Amélie Raba Léon, CHU de Bordeaux, Bordeaux, France; Sorbonne Université, INSERM, Institut Pierre Louis d’Epidémiologie et de Santé Publique (IPLESP), Equipe Sociale Santé Mentale et Addictions (ESSMA), Paris, France; Université Paris Cité and Université Sorbonne Paris Nord, Inserm, INRAE, Center for Research in Epidemiology and StatisticS (CRESS), Paris, France; Université Claude Bernard Lyon 1, CNRS, INSERM, Centre de Recherche en Neurosciences de Lyon (CRNL), U1028 UMR5292, Bron, France; Sorbonne Université, INSERM, Institut Pierre Louis d’Epidémiologie et de Santé Publique (IPLESP), Equipe Sociale Santé Mentale et Addictions (ESSMA), Paris, France; Sorbonne Université, INSERM, Institut Pierre Louis d’Epidémiologie et de Santé Publique (IPLESP), Equipe Sociale Santé Mentale et Addictions (ESSMA), Paris, France; Sorbonne Université, INSERM, Institut Pierre Louis d’Epidémiologie et de Santé Publique (IPLESP), Equipe Sociale Santé Mentale et Addictions (ESSMA), Paris, France; Sorbonne Université, INSERM, Institut Pierre Louis d’Epidémiologie et de Santé Publique (IPLESP), Equipe Sociale Santé Mentale et Addictions (ESSMA), Paris, France; Sorbonne Université, INSERM, Institut Pierre Louis d’Epidémiologie et de Santé Publique (IPLESP), Equipe Sociale Santé Mentale et Addictions (ESSMA), Paris, France; Université de Paris, INSERM, INRAE Centre for Research in Epidemiology and Statistics Paris, Paris, France; Ined Inserm EFS Joint Unit ELFE, Paris, France; Sorbonne Université, INSERM, Institut Pierre Louis d’Epidémiologie et de Santé Publique (IPLESP), Equipe Sociale Santé Mentale et Addictions (ESSMA), Paris, France

## Abstract

To examine the association between intimate partner psychological violence (P-IPV) from before pregnancy to 2 years after the child’s birth and child’s sleep patterns, i.e. sleep onset difficulty (SOD), nighttime awakenings (NA), and nighttime sleep duration (NSD) between 2 and 5 years of child’s age. Data come from the population-based French birth ELFE cohort launched in 2011. P-IPV was assessed before and during pregnancy, at 2 months and 2 years post-partum. Children’s sleep patterns were measured at 2, 3, and 5 years of age. Group-based trajectory modelling was used to identify trajectories of P-IPV and each child’s sleep patterns. Associations between P-IPV and children’s sleep trajectories were assessed by weighted multivariate logistic regressions. Five P-IPV trajectories were identified: minimal (64%), prenatal (14%), decreasing (9%), increasing (8%), and persistent (5%). Two trajectories of SOD (few 65% and many 35%), three trajectories of NA (few 49%, decreasing 24%, and many 23%), and three trajectories of NSD (short 21%, medium 56%, and long 23%) were identified. About 9513, 9512, and 9499 children were included in comparative analyses, respectively, focused on SOD, NA, and NSD. Increasing and persistent P-IPV trajectories were both associated with the trajectory of many SODs [odds ratio (OR) = 1.53, 95% confident interval (CI) = 1.24–1.91; and OR = 1.71, 95% CI = 1.31–2.22, respectively] and the trajectory of many NA (OR = 1.66, 95% CI = 1.29–2.13); and (OR = 1.95, 95% CI = 1.42–2.69, respectively). Associations between persistent P-IPV and decreasing and many NA were significant among girls (OR = 1.76, 95% CI = 1.12–2.75 and OR = 2.27, 95% CI = 1.39–3.71, respectively), but not among boys. Family interventions in response to IPV should pay particular attention to sleep patterns of children exposed to IPV.

## Introduction

Intimate partner violence (IPV) is a serious global public health problem. Nearly one-third of women between the ages of 15 and 49 have experienced IPV [1]. About 10.6% of men experienced contact sexual violence, physical violence, and/or stalking by an intimate partner in their lifetime, according to nationally representative data collected in the USA in 2015 [2]. Children exposed to IPV are co-victims and have important negative short- and long-term consequences for their mental and physical health, such as under-immunization or internalizing and externalizing behaviour problems in the short term and more risk-taking behaviours or substance abuse in adulthood [3, 4]. In a meta-analysis conducted in the UK, IPV exposure was the most prevalent form of child maltreatment, estimated at around 11.90% [5]. Among different types of IPV, intimate partner psychological violence (P-IPV) is the most common [6]. Among women, the prevalence rate of P-IPV was estimated at around 27% [6]. Psychological violence very often accompanies other types of violence. In the study conducted by Barbier *et al.* [7], physical or sexual IPV was isolated in <1% of cases.

Poor sleep quality is a precursor to further negative health outcomes, such as poor mental health and poor academic performance [8, 9]. Healthy sleep is often characterized by sufficient duration, good quality, and the absence of sleep disturbances such as difficulty in initiating or maintaining sleep [9]. Among the long-term consequences of sleep disruptions are metabolic disorders, including obesity and type 2 diabetes mellitus [9].

While several studies explored the association between IPV exposure during childhood and subsequent sleep disruptions [10–12], few studies focused on sleep problems before adulthood. Among these studies, adolescence was the most commonly studied period, and very few studies were conducted in childhood [10–14]. More difficulty in falling asleep, more difficulty in staying asleep, shorter sleep duration, and more frequent irregular bedtimes were reported among adolescents exposed to IPV during childhood [10–12]. In early childhood, Smith *et al.* [13] showed no association between P-IPV exposure at ages 3 to 5 and sleep problems at age 5. However, the sample of participants in this study included only women who experienced additional social adversity and the sample size was limited (*n* = 194). Similarly, in the study of Gustafsson *et al.*, including 185 children, no association was found between inter-parental verbal aggression exposure at 30 months and developmental trajectories of sleep problems between 24 and 36 months [14]. In contrast, physical IPV was associated with more sleep problems [14].

The objective of the current study was to explore, in a large population-based birth cohort, P-IPV trajectories from pre-pregnancy to 2 years after birth and child sleep problems between 2 and 5 years of age. Considered child sleep problems were nighttime awakenings (NA), sleep onset difficulty (SOD), and nighttime sleep duration (NSD) between 2 and 5 years of child’s age. It was hypothesized that persistent P-IPV during pregnancy and early childhood would be associated with more adverse child sleep trajectories, i.e. more SOD, more NA, and shorter NSD.

## Methods

### Study design and participants

Data were drawn from the French population-based ELFE cohort study (French Longitudinal Study since Childhood) [15]. A total of 320 randomly selected maternity units in metropolitan France participated in the year 2011. Inclusion criteria were infants born at ≥33 weeks of gestation, who were singletons or twins, for whom the family did not plan to leave metropolitan France within 3 years, and an informed consent from the mother with information to the father of his right to oppose. Infants born to mothers younger than 18 years of age who did not read French, Arabic, Turkish, or English were excluded. Due to the specificity of their sleep and the correlation between their data, twins were excluded from this study.

### Outcome

Parents were asked to report on their children’s sleep characteristics at 2, 3, and 5 years of age in phone interviews. Bedtime and wake time were assessed for weekdays and weekend days. A daily average NSD was estimated. Parents reported whether their child wakes up during the night and, if so, the number of nights this had happened in the past week. A categorical variable was created for NA from never, one to two nights per week, to more than two nights per week. SOD was also collected with the question: ‘When you put your child to bed, does he/she have difficulty falling asleep? For example, does he/she cry or fuss for more than 30 minutes?’ The response was never, sometimes, or often.

### Main exposure

Parental conflicts were collected from both parents (i) for the period before pregnancy, during pregnancy, and the first 2 months of child’s life, at the 2-month telephone interview, and (ii) during the 2-year telephone interview. Parents were asked to rate the frequency of arguments with their partner on a 4-point scale from ‘never’, ‘rarely’, ‘sometimes’, and ‘often’. If they reported arguing often or sometimes, they were asked to rate whether insults or hurtful comments were frequent (‘Has your partner ever insulted you or said hurtful things to you?’). The responses of both parents were combined. The maximum response was chosen if their responses differed. Thus, a variable with a 4-point scale was created for each period: ‘no insults or hurtful comments or never or rarely arguments’, ‘rarely’, ‘sometimes’, and ‘frequent insults or hurtful comments during arguments’. Single parents were grouped in the ‘no insults or hurtful comments or no or rare arguments’ category.

### Covariates

Known risk factors of IPV and variables that have been known to be associated with children’s sleep disorders were identified in the scientific literature [16–18]. The variables measured in the cohort were thus selected. A directed acyclic graph was used to identify covariates in adjusted models (Supplementary Fig. S1). Sociodemographic, parental, and child characteristics were collected at 2 months: Grand-parental birthplace, parental age, parental educational level, monthly household income in euros per consumption unit, parental employment, parental desire of pregnancy, whether the parents lived together or not, and the number of siblings. Maternal alcohol consumption during pregnancy was assessed in a face-to-face interview at the maternity ward. The child’s biological sex, prematurity, and low birth weight (defined as birth weight below the 10th percentile for gestational age) were extracted from children’s medical health records. We used standardized French intrauterine growth curves to determine low birth weight [19]. Parental smoking status and the main day-care arrangement were collected by phone interview at 1 year of age.

When the child was 2 years old, the following information was collected through telephone interviews with the mother and the father: The parent’s perception of grandparental support during the child’s second years, the frequency of the child’s presence during arguments between parent and his/her partner, ranging on a 4-point scale from ‘never’ to ‘often’. The responses with the highest frequency were selected if parents reported different frequencies. The mental health of both parents was assessed through the 6-item Kessler Psychological Distress Scale. This scale is a self-report questionnaire that screens global psychological distress over the past 30 days, with a range of 0 to 24. A score equal or greater than 13 indicates the probability of serious mental illness. The scale has been validated around the world and a French version showed good performances [20–22]. The child’s risk of autism was assessed by the Revised-Modified Check List Autism for Toddler. The parents completed the 20-item questionnaire from which the child was classified into three categories of risk: few, moderate, and high risk. The sensibility of the test was around 83%, and the specificity was 95% [23]. The questionnaire has been validated in French [24]. Parents reported the average time their child spent on each of five screens—TV, smartphone, video game console, tablet, and computer—during a typical weekday and weekend day. The average daily screen use over the week was the sum of reported screen time weighted by the times on weekdays and weekends [(weekdays × 5) + (weekend × 2)]/7 [25]. Finally, sleep routine was collected through several questions to the mother: The parent or caregiver who put the child to bed, the parent or caregiver who gets up at night when the child wakes up, the child falls asleep in own bed or not, and the child ends sleep in parent’s bed or not when the child wakes up at night.

### Statistical analyses

We performed group-based trajectory modelling using the latrend package in R to identify different longitudinal trajectories of P-IPV from before pregnancy up to the child’s 2 years of age and different longitudinal trajectories of average NSD, SOD, and NA between 2 and 5 years of the child’s age [26]. Group-based trajectory modelling handles missing values; however, children with no information or with only one point of measurement were excluded from these analyses. The Bayesian information criterion, the Weighted Mean Absolute Error, and the logLik were used to select the best-fitting models. We performed the selections via a manual elbow method, using the plotMetric function because of the consistent improvements of these criteria for an increasing number of clusters leading to an overestimation.

A specific weight was calculated for each participating child to deal with selection and attrition biases. Weights were calculated to take into account the sampling plan, initial non-response, and attrition during follow-up. To make ELFE participants nationally representative on variables such as age, region, marital status, migration status, level of education, and primiparity, National Statistical Data and the 2010 French National Perinatal study were used [27, 28].

Missing data on covariables were imputed using multiple imputation models by chained equations utilizing classification and regression trees assuming missing data at random [29]. The imputed datasets were used in weighted multivariate logistic regressions to compare the different trajectories of average NSD, difficulty falling asleep, and NA relative to the trajectories of P-IPV. Combined variables with information from both parents were created for pregnancy intention and parental education due to collinearity between information from the mother and father. Similarly, mother’s age was chosen to represent parental age. According to the DAG, minimally adjusted models (including household income, mother’s age, parental education level, parental unwanted pregnancy and immigrant status) and fully adjusted models (including in addition: Number of siblings, child’s biological sex, prematurity, low birth weight, risk of autism, alcohol use during pregnancy, main day-care arrangement, parental tobacco use, sleeping routine, parental mental health, parental employment, child’s presence during arguments) were performed (Supplementary Fig. S1). Interactions between the child’s biological sex and P-IPV exposure were explored. Sensitivity analyses were performed with complete case data.

### Ethics

The ELFE study has been approved by the authorities in accordance with the Declaration of Helsinki (Comité de Protection des Personnes [CPP in 2011 no. IDFIX-11-024]; Comité National Informatique et Libertés [CNIL no. 910504, no. 913074, no. 915770]; and CNIS [no. 2011X716AU, no. 2013X719AU, no. 2016X718AU]).

## Results

At birth, 18 329 children were included in the ELFE cohort. Finally, 9513 children followed for 5 years were included in the comparative analyses for SOD trajectories, 9512 for NA trajectories, and 9499 for NSD trajectories (Supplementary Fig. S2).

### P-IPV and sleep trajectories

**Figure 1.**
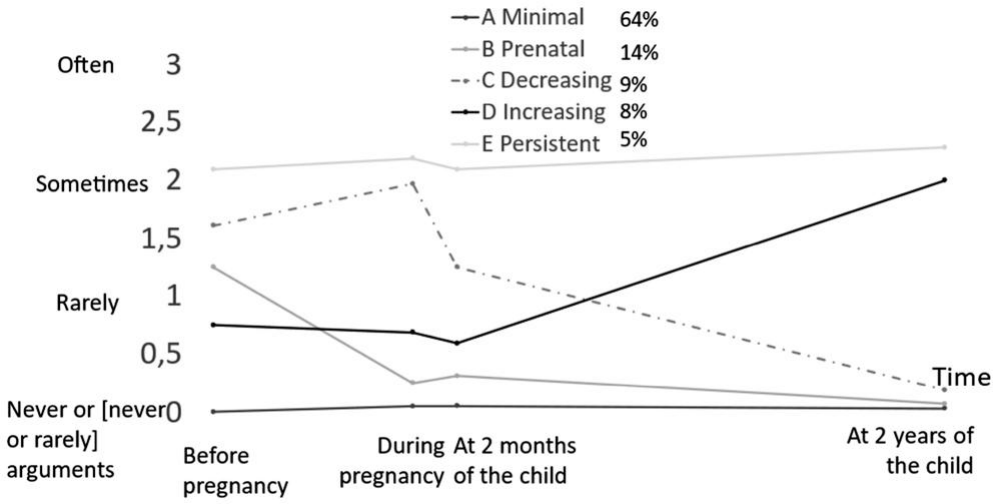
Description of the intimate partner psychological violence trajectories (insults or hurtful comments).

Five trajectories of P-IPV were identified (Fig. 1): A, minimal (64%), B, prenatal (14%), C, decreasing, P-IPV decreases after the child’s birth (9%), D, increasing (8%), P-IPV increases at 2 years of the child, and E, persistent (5%), always P-IPV.

**Figure 2.**
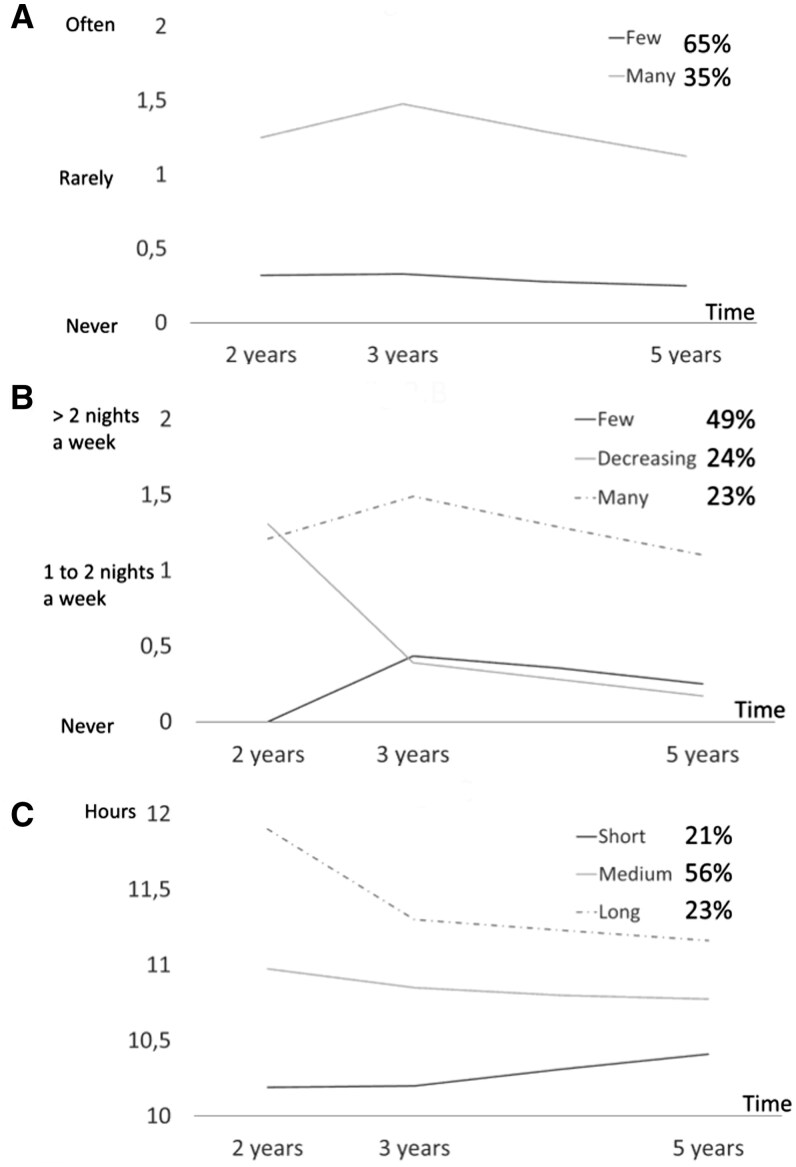
Description of sleep trajectories. (A) Sleep onset difficulty; (B) night awakening; and (C) night sleep duration.

We identified two trajectories of SOD (Fig. 2): few difficulties in falling asleep (65%) and frequent difficulties in falling asleep (35%); three trajectories of NA: few night awakenings (49%), decreasing night awakenings (24%), and many persistent night awakenings (23%); three trajectories of NSD: Short (21%), medium (56%), and long (23%).

### Description of the study population

Table 1 describes the characteristics of participating children and their families overall and according to the different sleep trajectories. The average daily duration of screen use at 2 years of age was longer among children on the trajectory with more SODs [median *M* = 0.36 h, interquartile range (IQR) = 0.11–0.71] than with few SODs (*M* = 0.30 h, IQR = 0.10–0.60). Similarly, the duration of screen use was more important among children with trajectories with more NA (*M* = 0.36 h, IQR = 0.10–0.70) for decreasing and *M* = 0.36 h, IQR = 0.13–0.69 persistent many than with few NA (*M* = 0.29 h, IQR = 0.08–0.57). When screens were used longer, sleep time was shorter: short (*M* = 0.36 h, IQR = 0.14–0.71), medium (*M* = 0.31 h IQR = 0.10, 0.64), and long sleep duration trajectories (*M* = 0.29 h, IQR = 0.08–0.61). The mean daily duration of screen use at 2 years of child’s age was longer in the persistent (*M* = 0.36 h, IQR = 0.13–0.71), decreasing (*M* = 0.36 h, IQR = 0.12–0.71), and increasing (*M* = 0.39 h, IQR = 0.14–0.71] P-IPV trajectories, than in the minimal (*M* = 0.29 h, IQR = 0.10–0.59) and prenatal (*M* = 0.29 h, IQR = 0.11–0.64) P-IPV trajectories. Similarly, children were less likely to fall asleep in their bed at 2 years in these P-IPV trajectories (Supplementary Table S1).

**Table 1.** Description of children participating in the French ELFE study from 2011 to 2016, and according to the trajectories of sleep between 2 and 5 yrs (*n* = 9513)

Variables	Miss	Trajectories (%)								Overall*N* = 9513
		Sleep onset difficulties		Night awakening			Night sleep duration			
		Few *N* = 6308	Many *N* = 3205	Few *N* = 4656	Decreasing *N* = 2685	Many *N* = 2171	Short *N* = 1978	Medium *N* = 5398	Long *N* = 2123	
Trajectories of intimate partner psychological violence	0									
A Minimal		66	58	66	62	58	61	64	63	63
B Prenatal		14	15	15	14	15	14	15	15	15
C Decreasing		9	11	9	10	10	10	9	10	9
D Increasing		7	10	6	9	10	9	7	9	8
E Persistent		4	6	4	5	7	6	5	3	5
*Child characteristics*										
Premature birth	138	4	4	4	4	5	4	3	5	5
Low birth weigh	258	7	7	6	7	8	8	6	8	7
Child’s biological sex: boy	73	50	51	50	51	52	58	50	46	50
Main day-care arrangement^a^	98									
Collective care		14	15	15	15	13	17	16	11	15
Cared by employed person		38	36	40	36	33	37	39	32	37
Cared by family members/partner		48	49	45	49	53	46	45	57	48
Child had his/her own bedroom^b^	51	77	74	78	75	73	71	76	79	76
Fall asleep in his/her own bed^b^	52	95	87	95	92	87	85	94	94	92
End of the night in the parents’ bed when night awaking^b^	197	19	36	7	38	43	39	24	15	25
Child’s presence during parental conflict^b^	3									
No or rare arguments		33	28	34	29	28	28	30	36	31
Rarely		33	31	32	35	30	31	33	32	32
Sometimes		30	34	29	31	37	34	32	28	32
Often		4	7	5	5	5	7	5	4	5
*Parental characteristics*										
Mother’s age (years)^c^	0									
<26		14	14	14	16	13	11	12	21	14
26–30		35	35	35	34	37	31	36	37	35
31–35		33	33	33	32	32	34	34	29	33
36–40		15	15	15	15	16	19	15	12	15
>40		3	3	3	3	2	5	3	2	3
Father’s age (years)^c^	30									
<26		9	8	9	10	7	6	8	12	9
26–30		28	28	27	28	29	25	28	31	28
31–35		33	31	32	31	32	31	33	32	32
36–40		19	21	20	20	19	22	20	16	20
>40		11	12	12	11	12	16	11	9	11
Mother’s migrant status^c^	17									
French with 2 parents French		78	71	77	77	70	66	78	79	75
French with 1 parent not French		6	6	6	6	7	6	6	5	6
French with 2 parents not French		3	5	4	4	5	5	3	4	4
Not native French		13	18	13	14	18	23	13	12	15
Father’s migrant status^c^	615									
French with 2 parents French		78	75	79	78	72	71	79	79	77
French with 1 parent not French		14	16	14	15	17	19	13	15	15
French with 2 parents not French		4	4	3	3	5	4	4	2	4
Not native French		4	5	4	4	6	6	4	4	4
Parental employment^c^	10									
Two parents unemployed		6	6	5	7	6	5	5	7	6
Two parents working		63	60	64	60	60	65	63	57	62
One parent unemployed		31	34	31	33	34	30	32	36	32
Mother’s educational level^c^	97									
Secondary school or less		47	47	46	47	49	45	44	55	47
First cycle of higher education		19	19	19	20	18	19	20	18	19
≥Second cycle		34	34	35	33	33	36	36	27	34
Father’s educational level^c^	413									
Secondary school or less		52	53	50	53	54	51	50	58	52
First cycle of higher education		18	16	19	17	17	18	18	16	18
≥Second cycle		30	31	31	30	29	31	32	26	30
Desire of pregnancy										
Mother’s hesitation or child unwanted	23	6	6	5	6	7	6	6	6	6
Father’s hesitation or child unwanted	64	10	10	9	11	11	10	10	10	10
At least one parent smoker^a^	693	44	48	44	45	47	39	44	53	45
Maternal psychological distress^b^	53	4	7	3	5	7	4	4	6	5
Paternal psychological distress^b^	955	2	2	1	2	2	2	2	2	2
*Familial characteristics*										
Parents not living together^c^	0	1	1	1	1	1	1	1	1	1
Number of siblings^c^	0									
0		40	45	40	43	43	43	40	43	41
1		38	35	38	35	37	34	39	35	37
2		16	15	17	15	14	16	16	15	16
≥3		6	5	5	7	6	7	5	7	6
Monthly household income by part (euros)^c^	234									
<1286		39	41	38	40	42	37	38	46	40
[1286, 1667)		29	28	28	30	29	28	29	28	29
[1667, 2083)		15	13	15	14	12	16	14	12	14
≥2083		17	18	19	16	17	19	19	14	17
Grand-parental support^b^	973									
Both parents answer yes		63	67	64	65	65	65	64	65	64
One parent answer yes		5	4	4	5	5	5	4	4	5
Both parents answer no		32	29	32	30	30	30	32	31	31
Parent who sleeps the child at night^b^	365									
Always the mother		14	15	13	15	17	16	13	15	14
Often the mother		32	31	30	31	33	33	32	28	31
Mother and partner		45	43	48	44	39	41	45	48	45
Often the partner		7	10	7	8	9	8	8	7	8
Always the partner		2	1	2	2	2	2	2	2	2
Another person		0	0	0	0	0	0	0	0	0

a At the age of 1 year.

b At the age of 2 years.

c At the age of 2 months of child.

### Association between sleep trajectories and P-IPV trajectories

Table 2 shows the results of multivariate analyses. P-IPV trajectories were associated with the trajectory of SOD, particularly increasing [odds ratio (OR) = 1.53, 95% confident interval (CI) = (1.24–1.91)] and persistent trajectories (OR = 1.71, 95% CI =  1.31–2.22). Children who were in the increasing and persistent P-IPV exposure group had a higher risk of being in the many persistent NA trajectory (OR = 1.66, 95% CI = 1.29–2.13) and OR = 1.95, 95% CI = 1.42–2.69, respectively). Increasing P-IPV exposure at 2 years of age was associated with the decreasing trajectory of SOD (OR = 1.49, 95% CI = 1.15–1.91).

**Table 2.** Association between intimate partner psychological violence trajectories and the trajectories of sleeping in the French ELFE study from 2011 to 2016: multivariate logistic regressions with imputed weight data

Variables	Sleep onset difficultiesOR (95% CI)	Night awakeningsOR (95% CI)				Night sleep durationOR (95% CI)		
	Many versus few	Decreasing versus few		Many versus few		Short versus medium	Long versus medium	Short versus long
	*N* = 9513	*N* = 7341		*N* = 6827		*N* = 7367	*N* = 7541	*N* = 4090
**Minimal adjusted models** ^a^								
Trajectories of P-IPV								
A Minimal	Reference	Reference		Reference		Reference	Reference	Reference
B Prenatal	1.23 (1.04–1.45)	0.95 (0.78–1.14)		1.11 (0.91–1.35)		0.97 (0.80–1.20)	1.02 (0.84–1.23)	0.96 (0.75–1.22)
C Decreasing	1.31 (1.07–1.59)	1.11 (0.89–1.39)		1.21 (0.96–1.54)		1.09 (0.87–1.38)	1.06 (0.84–1.34)	1.03 (0.78–1.36)
D Increasing	1.53 (1.24–1.91)	1.49 (1.15–1.91)		1.66 (1.29–2.13)		1.33 (1.02–1.73)	1.25 (0.96–1.60)	1.10 (0.80–1.50)
E Persistent	1.71 (1.31–2.22)	1.34 (0.98–1.84)		1.95 (1.42–2.69)		1.33 (0.97–1.80)	0.66 (0.44–1.98)	2.05 (1.34–3.14)
**Full adjusted models** ^b^		Among girls	Among boys	Among girls	Among boys			
Trajectories of P-IPV								
A Minimal	Reference	Reference	Reference	Reference	Reference	Reference	Reference	Reference
B Prenatal	1.17 (0.98–1.39)	0.88 (0.67–1.15)	0.94 (0.71–1.22)	0.93 (0.69–1.25)	1.11 (0.84–1.46)	0.94 (0.76–1.16)	1.12 (0.92–1.36)	0.87 (0.67–1.12)
C Decreasing	1.22 (0.99–1.49)	1.23 (0.89–1.69)	0.91 (0.67–1.23)	1.22 (0.85–1.74)	0.96 (0.69–1.33)	1.06 (0.83–1.35)	1.15 (0.90–1.46)	0.93 (0.69–1.25)
D Increasing	1.30 (1.03–1.64)	1.49 (1.04–2.12)	1.20 (0.83–1.71)	1.55 (1.04–2.28)	1.29 (0.89–1.86)	1.22 (0.92–1.62)	1.42 (1.09–1.84)	0.85 (0.59–1.22)
E Persistent	1.39 (1.05–1.85)	1.76 (1.12–2.75)	0.94 (0.59–1.48)	2.27 (1.39–3.71)	1.18 (0.76–1.83)	1.08 (0.77–1.49)	0.78 (0.52–1.17)	1.39 (0.89–2.15)

a Adjusted on household income, mother’s age, parental education level, parental unwanted pregnancy, and immigrant status.

b Adjusted on household income, mother’s age, parental education level, parental immigrant status, number of siblings, child’s biological sex, consumption of alcohol during pregnancy, Modified check list Autism for Toddler at 2 years of the child, prematurity, low birth weight, parental mental health, parental employment, main day-care arrangement at the age of 1 year, at least one parent smoker or not at the age of 1 year, sleeping routine at the age of 2 years, and child’s presence during arguments at the age of 2 years.

### Interactions between child’s biological sex and P-IPV trajectories

Interaction tests between the child’s biological sex and P-IPV trajectories were not statistically significant in fully adjusted and bivariate statistical models for SOD and NSD. Associations between P-IPV exposure and SOD and NSD were statistically similar between boys and girls. Interaction tests between the child’s biological sex and P-IPV trajectories were statistically significant for NA. Associations between persistent P-IPV and NA were statistically significant among girls [decreasing vs. few NA (OR = 1.76, 95% CI  = 1.12–2.75); many vs. few NA (OR = 2.27, 95% CI = 1.39–3.71, respectively], but not among boys (Table 2).

## Discussion

The present study examined the association between early P-IPV exposure and children’s sleep patterns in a large population-based birth cohort. Very few studies focus on the first years of life. We observed that persistent P-IPV up to 2 years of age was associated with greater SOD and staying asleep. Children with persistent P-IPV exposure also had shorter NSD, but the association did not persist after adjustment for potential variables on the causal pathway. To a lesser extent, the same was observed for SOD and prenatal and decreasing P-IPV trajectories. The effect of P-IPV appears to be more important for girls than for boys, concerning NA, which is partly consistent with previous studies among adolescents [11].

These results are not consistent with previous studies in the childhood [13, 14] but are in line with studies conducted during adolescence [10–12]. There could be several explanations for this. First, in the study of Smith *et al.*, P-IPV exposure was measured between the ages of 3 and 5 years, and different trajectories over time were not examined. Children were classified as exposed or not exposed without distinguishing variations in exposure over time. Second, in the same study, sleep problems were assessed with one question with 5-year-olds (problems with sleep pattern or habits) [13]. In this study, we showed that different characteristics of children’s sleep were not associated in the same way with P-IPV exposure, and, in particular, we found less association with NSD. Additionally, parental perception of the child’s sleep problems could be different according to many complex factors, including culture and parental beliefs and cognitions [30]. In the second study, the sample size was probably too small (*n* = 185) to show a significant difference between groups [14].

The possible explanations of the effects of P-IPV exposure on children’s sleep are many and complex, as suggested by several models, including the transactional model of Sadeh and Anders [30, 31]. In this model, children need to develop self-regulation skills to soothe themselves and fall asleep independently of their parents. However, cultural, environmental, and family factors influence intrinsic child and parental factors, which, in turn, influence the parent–child interaction, strongly linked to the child’s sleep patterns [31]. In this context, P-IPV would increase the victim parent’s separation anxiety, with an over-sensitivity to the child’s distress signal and more guilt and sadness when separated from her child during sleep. This anxiety could affect parental nighttime involvement and, thus, the child’s ability to self-regulate and soothe. Similarly, parental emotional availability and positive feelings could be less important for parents experiencing P-IPV, particularly through the effect on parental mental health. Furthermore, going to sleep represents a separation between the child and his/her attachment figure. Noonan *et al.* [32] showed that IPV exposure during childhood is associated with less secure child–parent attachment for the child. Children exposed to P-IPV could have more sleep disturbances through this mechanism. Regarding the intrinsic child factor, several studies have already shown that children exposed to P-IPV have more emotional problems than those who are not exposed [13, 33, 34]. The relationship between sleep and emotional and behavioural functioning is strong and probably bidirectional [35]. Children exposed to P-IPV could have more emotional problems, which could affect their development of self-regulation skills.

It is difficult to explain why girls are more likely to wake up at night when they are exposed to P-IPV, while boys are not. A systematic review of the prevalence of nightmares in children found that the prevalence of nightmares was higher in girls than boys [36]. Nightmares could be responsible for night awakening. The consequences of exposure to IPV could also differ depending on the child’s biological sex. Women are more often the victims of IPV, and daughters could be more likely to identify with their mothers, which could have a greater impact on their sleep patterns. Finally, a recent study shows that women are more empathetic than men, which could also be an explanation of why parental conflicts have more of an impact on girls [37].

This study has several strengths. We used data from a large nationwide sample. The prospective and longitudinal design is particularly well suited to better understand possible causal pathways between exposure to violence and children’s sleep patterns. In addition, we collected information about potential confounding factors.

This study also has some limitations. First, only P-IPV was measured in our study. P-IPV is the most common form of IPV and is present in the vast majority of other forms of IPV [7]. However, the simultaneous presence of multiple forms of violence increases the severity of the situation. The combined effects of multiple violence types are, therefore, not considered in our study. When P-IPV is persistent over time, the prevalence of other forms, such as physical violence, could be more important and responsible for the significant impact on the child. Second, the child’s sleep characteristics were reported by their parents. Parents’ perceptions of SOD or NA could differ according to their interpretation. Parents who are depressed or experience significant fatigue could perceive their child’s sleep problems as more severe. This could increase the estimated association between sleep characteristics and P-IPV exposure because victims of IPV are more likely to have poor mental health in the minimal adjusted models [4]. The full-adjusted models included parental mental health. Third, NSD was probably overestimated because it was calculated based on bedtime and waking time, but the child may fall asleep later. We did not use standardized validated questionnaires to assess the P-IPV exposure. In the same way, the interpretation of ‘insults or hurtful comments’ and the frequency of P-IPV exposure (‘never’ to ‘often’ without precise quantification) could be different according to the parental characteristics. When parents are tired, this could make them more susceptible. Furthermore, socially desirable responses may lead to underreporting of involvement in partner violence. Victims and authors may not report the violence to avoid feeling shame and guilt. Although we collected a comprehensive set of confounding variables, there are likely to be other unmeasured factors that would be interesting to investigate, such as other types of child maltreatment and sleep-disordered breathing. Finally, cultural context influences the parental sleep-related practices, daily parenting practices, and probably parental perception of their child’s sleep problems [31]. Even if we took into account sleep routine and the parental immigrant status in our study, some cultural aspects could be responsible for our findings not being generalizable to all cultural contexts.

These findings highlight how early persistent and increasing P-IPV exposures are related to children’s difficulties in falling and staying asleep between 2 and 5 years of age. Clinicians and professionals involved in caring for children who experience family violence should pay special attention to children’s sleep. Several interventions to improve sleep quality in children have been developed, including healthy sleep practices [38, 39]. In the context of IPV, it could be more difficult to support parents to adopt these practices and to ensure a positive, calm, and relaxed atmosphere in the child’s environment. Specific interventions for families at risk, including domestic violence, have been developed and may be of interest [40]. However, the first and probably indispensable element in ensuring the effectiveness of interventions in this context must be to prevent the child from being exposed to IPV.

## Supplementary Material

ckaf037_Supplementary_Data

## Data Availability

Data of the study are protected under the protection of health data regulation set by the French National Commission on Informatics and Liberty (Commission Nationale de l’Informatique et des Libertés, CNIL). Deidentified individual participant data (including data dictionaries) will be made available, in addition to study protocols, the statistical analysis plan, and the informed consent form. The data will be made available upon publication to researchers who provide a methodologically sound proposal for use in achieving the goals of the approved proposal. Proposals should be submitted to the authors. Key points Sleep quality is a precursor of further negative health outcomes. Exposure to intimate partner violence (IPV) in childhood is associated with more sleep problems in adulthood and probably adolescence. Persistent and increasing psychological-IPV before 2 years of age was associated with more difficulties in falling asleep and frequent NA between 2 and 5 years of age. Associations between psychological IPV and frequent night awakening were stronger among girls than boys.
