# Supplementary material for: Intimate partner psychological violence and children’s sleep difficulties up to 5 years of age: an ELFE birth cohort
Source: Eur J Public Health. 2025 Apr 4;35(5):873–81. doi: 10.1093/eurpub/ckaf037 (PMC12529263; doi:10.1093/eurpub/ckaf037)
Supplement: ckaf037_Supplementary_Data [file ckaf037_supplementary_data.zip › ckaf037_Supplementary_Data/ejph-2024-12-om-0912-File006.docx]

**Table S1** Description of children participating in the French ELFE study from 2011 to 2016, and according to the trajectories of P-IPV (n=9513)

| **Variables** | **Miss** | **Trajectories of Intimate Partner Psychological Violence (%)** | | | | | | **Overall**  **9513** |
| --- | --- | --- | --- | --- | --- | --- | --- | --- |
|  |  | **A minimal**  **6125** | **B**  **Prenatal**  **1401** | **C**  **Decreasing**  **866** | **D**  **Increasing**  **704** | **E**  **Persistent**  **417** |  | |
| Trajectories of sleep onset difficulties*  Few difficulties  Difficulties | 0 | 68  32 | 63  37 | 61  39 | 58  42 | 54  46 | | 65  35 |
| Trajectories of night awakening*  Few  Decreasing  Many | 1 | 50  28  22 | 49  26  25 | 45  29  26 | 38  33  29 | 36  29  35 | | 48  28  24 |
| Trajectories of night sleep duration*  Short  Medium  Long | 14 | 21  55  24 | 20  55  25 | 22  53  25 | 24  48  28 | 28  55  17 | | 21  55  24 |
| Premature birth | 138 | 4 | 4 | 4 | 5 | 5 | | 4 |
| Low birth weigh | 258 | 7 | 8 | 6 | 6 | 9 | | 7 |
| Child’s gender: boy | 73 | 50 | 51 | 51 | 49 | 54 | | 51 |
| **At two months of the child** |  |  |  |  |  |  | |  |
| Mother’s age (years)*  <26  26-30  31-35  36-40  >40 | 0 | 13  36  33  15  3 | 14  37  33  14  2 | 15  34  36  13  2 | 22  30  32  13  3 | 15  33  32  17  3 | | 14  35  33  15  3 |
| Father’s age (years)*  <26  26-30  31-35  36-40  >40 | 30 | 7  29  32  20  12 | 10  30  31  18  11 | 9  23  36  21  11 | 16  25  28  20  11 | 12  28  31  18  11 | | 9  28  32  20  11 |
| Mother’s migrant status*  French with 2 parents French  French with 1 not French  French with 2 not French  Not native French | 17 | 77  5  3  15 | 75  6  3  16 | 72  8  5  15 | 76  7  7  10 | 66  8  6  20 | | 75  6  4  15 |
| Father’s migrant status  French with 2 parents French  French with 1 not French  French with 2 not French  Not native French | 615 | 78  14  4  4 | 77  15  5  3 | 74  17  5  4 | 76  15  4  5 | 72  18  5  5 | | 77  15  4  4 |
| Parental employment*  Two parents unemployed  Two parents working  One parent unemployed | 10 | 5  64  31 | 4  62  34 | 6  58  35 | 11  57  32 | 10  51  39 | | 6  62  32 |
| Parents living not together | 0 | 1 | 0 | 2 | 3 | 1 | | 1 |
| Number of siblings * 0  1  2  ≥3 | 0 | 42  36  16  6 | 42  38  15  5 | 34  41  17  8 | 46  34  15  5 | 34  42  16  8 | | 42  37  15  6 |
| Monthly household income by part (euros)* <1286  [1286,1667)  [1667, 2083)  ≥2083 | 234 | 38  29  15  18 | 37  30  14  19 | 46  26  12  18 | 44  29  12  15 | 50  26  12  12 | | 40  29  14  17 |
| Mother’s educational level*  Secondary school or less  First cycle of higher education  ≥Second cycle | 97 | 45  19  36 | 47  20  33 | 47  22  31 | 56  18  26 | 53  14  33 | | 47  19  34 |
| Father’s educational level*  Secondary school or less  First cycle of higher education  ≥Second cycle | 413 | 51  18  31 | 50  20  30 | 54  17  29 | 58  15  27 | 58  15  27 | | 52  18  30 |
| Desire of pregnancy  Mother’s hesitation or child unwanted  Father’s hesitation or child unwanted* | 23  64 | 6  8 | 5  11 | 7  12 | 8  13 | 7  21 | | 6  10 |
| Mother’s alcohol consumption during pregnancy  Never when pregnancy known  Max one time per month  More than one time per month | 104 | 82  17  1 | 81  18  1 | 81  17  2 | 77  19  4 | 76  22  2 | | 81  17  2 |
| **Age one years** |  |  |  |  |  |  | |  |
| At least one parent smoker* | 693 | 42 | 45 | 51 | 55 | 55 | | 45 |
| Main day-care arrangement at age 1 year*  Collective year  Cared by employed person  Cared by family | 25 | 14  39  47 | 17  38  45 | 15  33  52 | 13  31  56 | 20  29  51 | | 15  37  48 |
| **Age two years** |  |  |  |  | | | |  |
| Maternal psychological distress* | 53 | 3 | 4 | 6 | 8 | 16 | | 5 |
| Paternal psychological distress* | 955 | 1 | 1 | 2 | 6 | 4 | | 2 |
| Modified-Check List Autism for Toddler M-CHAT*  Low risk (0-2 failures)  Medium risk (3-7 failures)  High risk (8 or more failures) | 2 | 87  13  0 | 84  16  0 | 88  12  0 | 85  15  0 | 83  17  0 | | 87  13  0 |
| Child’s presence during parental conflict*  No or rare arguments  Rarely  Sometimes  often | 3 | 41  33  24  2 | 17  39  40  4 | 19  31  45  5 | 6  22  53  19 | 13  16  48  23 | | 31  32  32  5 |
| Grand-parental support  Both parents answer yes  One parent answer yes  Both parents answer no | 973 | 65  31  4 | 64  31  5 | 64  31  5 | 66  31  3 | 60  32  8 | | 64  31  5 |
| Child had his/her own bedroom* | 51 | 78 | 75 | 68 | 75 | 69 | | 76 |
| Fall asleep in his/her own bed* | 52 | 93 | 93 | 89 | 89 | 84 | | 92 |
| End of the night in the parental bed when night awaking* | 197 | 23 | 23 | 30 | 28 | 36 | | 25 |
| Parent who sleeps the child at night*  Always the mother  Often the mother  Mother and partner  Often the partner  Always the partner  Another person | 365 | 13  30  47  8  2  0 | 13  34  43  9  1  0 | 16  35  39  8  2  0 | 20  32  38  8  2  0 | 21  32  34  12  1  0 | | 15  31  45  8  1  0 |
| Parent who wakes up at night to put the child back to sleep*  Always the mother  Often the mother  Mother and partner  Often the partner  Always the partner  Another person  Not concerned | 365 | 26  28  33  9  3  0  1 | 25  28  31  11  3  0  2 | 29  32  26  9  2  0  2 | 32  26  28  11  1  0  2 | 28  32  26  9  4  0  1 | | 26  28  31  10  3  0  2 |

*p-value < 0.05 with Wilcoxon rank-sum test for complex survey samples; chi-squared test with Rao & Scott’s second-order correction
